# Novel Nano-Precursor Inhibiting Material for Improving Chloride Penetration Resistance of Concrete: Evaluation and Mechanism

**DOI:** 10.3390/ma14205929

**Published:** 2021-10-09

**Authors:** Ruixing Chen, Song Mu, Jiaping Liu, Jingshun Cai, Deqing Xie, Guangyan Liu, Zheng Guo

**Affiliations:** 1 School of Materials Science and Engineering, Southeast University, Nanjing 211189, China; crx31824@163.com; 2 State Key Laboratory of High Performance Civil Engineering Materials, Jiangsu Research Institute of Building Science, Nanjing 211103, China; caijingshun@cnjsjk.cn (J.C.); xiedeqing@cnjsjk.cn (D.X.); liuguangyan@cnjsjk.cn (G.L.); guozheng@cnjsjk.cn (Z.G.); 3 Jiangsu SOBUTE New Materials Co., Ltd., Nanjing 211103, China

**Keywords:** chloride transport, hydrophobic, concrete, nanomaterials, pore structure

## Abstract

Durability improvement is always important for steel–concrete structures exposed to chloride salt environment. The present research investigated the influence of a novel nano-precursor inhibiting material (NPI), organic carboxylic acid ammonium salt, on the mechanical and transport properties of concrete. The NPI caused a slight reduction in the strength of concrete at later ages. NPI significantly decreased water absorption and slowed down the speed of water absorption of concrete. In addition, the NPI decreased the charge passed and the chloride migration coefficient, and the results of the natural chloride diffusion showed that the NPI decreased the chloride concentration and the chloride diffusion coefficient. The NPI effectively improved the resistance of chloride penetration into testing concrete. The improvement in the impermeability of concrete was ascribed to the incorporation with the NPI, which resulted in increasing the contact angle of cement pastes. The contact angle went up from 17.8° to 85.8° for 0% and 1.2% NPI, respectively, and cement pastes became less hydrophilic. Some small pore throats were unconnected. Besides, the NPI also optimized the pore size distribution of hardened cement paste.

## 1. Introduction

Chloride salt-induced corrosion of reinforcement steel, in concrete structures exposed to chloride environments, has caused huge losses and casualties. The durability of reinforced concrete remains a challenge for developing green and sustainable infrastructures [1].

Chloride diffusion coefficient is a key parameter of concrete that closely relates with rebar depassivation. Traditionally, many approaches have been applied to decrease the chloride diffusivity of cement-based materials or increase the time of rebar depassivation, including lowering the water–cement ratio of the concrete, replacing cement with mineral admixtures [2], expanding the thickness of cover, and using some nanomaterials [3,4,5,6] and polymers [7,8,9,10,11]. Besides, there are other methods to improve the durability of the concrete structures: for example, Yu et al. [12] applied bidirectional electromigration rehabilitation technology to improve the durability of chloride-contaminated concrete structures. However, lowering the water–cement ratio and adding mineral admixtures can increase the viscosity and autogenous shrinkage of concrete in practical engineering applications. The application of nanomaterials should solve the effective dispersion problem in concrete, and polymers could cause huge loss of strength.

Some new chemical admixtures were reported to decrease the water absorption and chloride diffusivity of concrete. Atzeni [13] reported that silicic esters and alkylalkoxysiloxane were used to promote the water-repelling ability of cement-based materials, leading to a clear reduction of water sorptivity. Aggarwal [14] found that 30% (by mass of cement) epoxy emulsion, produced with diglycidyl ether of bisphenol-A and amino-amide-based hardener, resulted in 45% and 55% reduction in water absorption, respectively, due to the porosity reduction of mortars. Franzoni [15] observed that the contact angle of the concrete sample increased from 36° to 48° on treating it with ethyl silicate, and substantial reduction of chloride penetrating into concrete was achieved. Holmes [16] also reported that the siloxane-based corrosion inhibitor mitigated the corrosion of steel, improved the steel corrosion resistance and water ingress by adding 4% (by mass of binder) inhibitor, but caused a reduction in compressive strength of ~12 MPa because of the pore structure change and the hydration inhibition. Zhang [17] revealed that polycarboxylate superplasticizer changed the dispersion of cement particles by exerting an electrostatic and/or steric force, which helped to form a denser microstructure. Poly-acrylate latexes adsorbed on the surface of cement grains, thus causing a better dispersion and consequently decreasing the pore size of cement paste. On the other hand, some polymer particles filled the capillary pores since particle size was in the range of 100–1000 nm, which increased the impermeability of cement-based materials. Zhou [18] manufactured polymers with different functional groups and investigated the repelling water effect. The results of molecular dynamics revealed that the polarity carboxyl group of polymers adsorbed on the CSH surface, while hydrophobic carbon chains played a role like a gate and had an inhibiting effect when the cement paste contacted with water [18]. Hou [3] found that tetraethoxysilane (TEOS) and nano-SiO_2_ were used to modulate the transport properties: TEOS decreased the porosity of the pores smaller than 0.1 μm, while nano-SiO_2_ blocked the pores of around 0.1 μm, and thus reduction in water absorption was achieved in the case of both. Kong [19] observed that tetraethoxysilane (TEOS) had a negative effect on the compressive strength of cement-based materials at all curing ages, 3-aminopropyltriethoxysilane (APTES) and N-2-aminoethyl-3-aminopropyltrimethoxysilane (AEAPTMS) enhanced the compressive strength after 3-day curing, three silanes showed a retardation in hydration, and the usage of silane increased both porosity and capillary pore volume, which resulted from a lower hydration degree and the air-entraining effect, while it should be noted that addition of AEAPTMS decreased the threshold pore size. Feng [20] investigated the effect of silanes with different functional groups on cement pastes and mortar by several methods: 3-aminopropyl triethoxysilane and 3-glycidyloxypropyl trimethoxysilane decreased the total porosity and their pore size distribution shifted to a smaller direction; vinyl triethoxysilane increased the total porosity and formed bigger pores; 3-aminopropyl triethoxysilane, vinyl triethoxysilane, and 3-glycidyloxypropyl trimethoxysilane enhanced strength, the last one increasing the compressive and splitting strength of mortar by 20% and 38%, respectively. Feng [21] found that mortar became hydrophobic by addition of stearic acid aqueous emulsion, the resistance of water and chloride penetration into concrete was enhanced, but the mechanical strength declined severely. Polymers were also used to modify the surface of cement-based materials [22,23,24,25,26], and the durability was improved [27,28,29,30,31]. Some hydrophobic metal soaps were also used to modify the properties of concrete by depositing hydrophobic layers on the pore surface, but there were doubts about their long-term effects. The effect of these polymers on water absorption, chloride resistance, and compressive strength was significantly different based on their functional group and dosage. There was urgent need to solve the severe strength loss problem and to figure out the actual chloride concentration when concrete with hydrophobic materials were used in chloride salt environments.

As one of the promising chemical admixtures, nanomaterials can be used to improve the durability performance of cementitious materials by refining the pore size distribution and accelerating hydration, while they may cause agglomeration and loss of workability because of their high specific surface. However, there is a strong demand for water-soluble and easy-to-use agents to enhance concrete resistance to chloride ion penetration. Based on preliminary research [32], the NPI can interact with the hydrated products of cement paste. The purpose of this research was to explore the effect of organic carboxylic acid ammonium salt on the properties of cement-based materials.

## 2. Materials and Methods

### 2.1. Materials

The chemical compositions of commercially available ordinary Portland cement (OPC, China united cement Co., Ltd. and China National Building Materials Group Co,. Ltd., Beijing, China, fly ash (FA), and ground granulated blast furnace slag (GGBS) are shown in Table 1.

**Figure 1:**
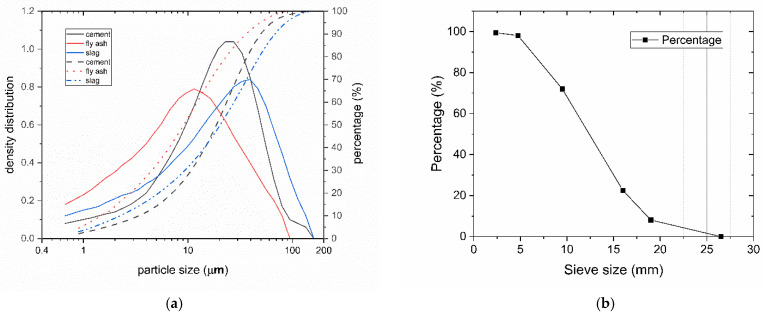
(**a**) Particle size distribution of cement, fly ash, and slag. Solid lines show the cumulative distribution, and dotted lines show the density distributions. (**b**) Gradation curve of coarse aggregates.

OPC was manufactured for testing concrete admixtures without mineral admixtures based on the Chinese standard Concrete admixtures (GB/T 8076-2008) [33]. The specific surface area of cement, fly ash, and slag are 2531 cm^2^/g, 4827 cm^2^/g, and 3036 cm^2^/g respectively. Their particle sizes distributions are illustrated in Figure 1. The particle size D_50_ and volume mean diameter are 18.68 µm and 23.97 µm for cement, 9.12 µm and 14.61 µm for fly ash, and 20.35 µm and 29.03 µm for slag, respectively. The range of particle size distribution of cement is narrower than that of fly ash and slag. Natural river sand and mechanically crushed stone were used in this experiment. The fineness modulus of river sand was 2.6. Figure 1b shows the gradation curve of coarse aggregates; the water absorption of coarse aggregates was 1.3%.

**Figure 2:**
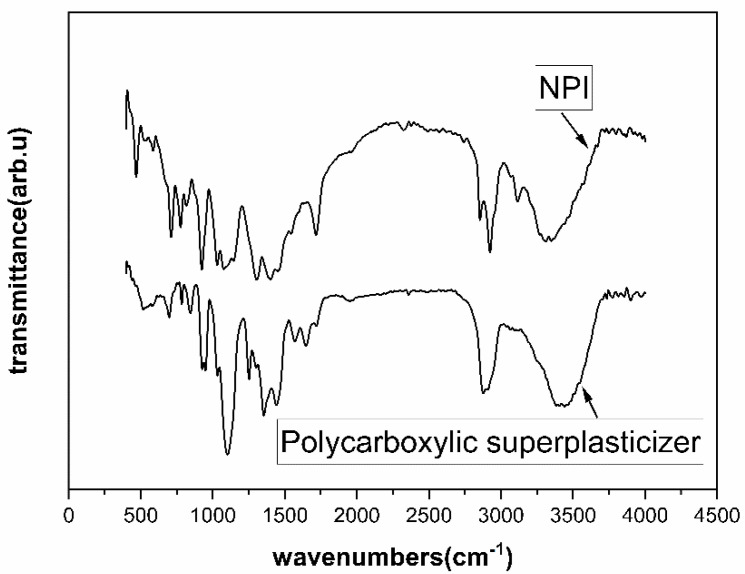
FT-IR spectra of polycarboxylic superplasticizer and nano-precursor inhibiting material (NPI).

The product of organic alcohol amine salt was a light yellow liquid product produced by Jiangsu Sobute New materials Co., Ltd., Nanjing, China. Its solid content was 18%. The preparation process was described as follows: aliphatic organic carboxylic acid with a carbon atom number of 8 to 22, alkylol amine, ethanol, and an appropriate catalyst were put into a three-necked flask and stirred evenly and heated to 140 °C for 12 h. Water and other functional additives were slowly added to the flask after heating treatment and cooling down. Finally, the required concentration of the nano-precursor inhibiting material (NPI) was prepared. The infrared spectrum of the NPI is shown in Figure 2. Weak absorption at 3650 cm^−1^ indicated the presence of small amounts of organic acids, and a clear absorption peak between 3300–3500 cm^−1^ indicated the existence of -NH_2_ or -NH groups. An absorption peak at 1380 cm^−1^ was further evidence of the presence of the above groups. An absorption peak at 3100 cm^−1^ indicated the subsistence of C-H, and an absorption peak at 2850–2960 cm^−1^ indicated the presence of -CH_2_. An absorption peak at 1000–1300 cm^−1^ indicated the existence of a large number of -C-O bonds.

### 2.2. Methods

#### 2.2.1. Specimen Preparation

The concrete mix is shown in Table 2. Considering practical engineering applications, the water-to-binder ratio of the concrete was 0.4, and the total amount of the binder was 490 kg/m^3^. Concrete specimens with 0%, 0.6%, 0.9%, and 1.2% NPI were cast in this study. The workability of concrete was adjusted by adding a polycarboxylic superplasticizer. The slump was 160 ± 20 mm. The samples were placed in a curing room (the temperature was 20 ± 5 °C, and relative humidity more than 95%) after demoulding.

#### 2.2.2. The Compressive Strength Test

The compressive strength of the concrete was tested at 3, 7, 28, and 112 days according to the China standard for test method of mechanical properties on ordinary concrete (GB/T 50081-2002) [34], which was similar to British standard BS EN 12390-3 [35]. The sample dimension was 100 mm × 100 mm × 100 mm, and a minimum of six specimens were used for the test. The load speed was 0.5~0.8 MPa/s.

#### 2.2.3. Isothermal Calorimetry

Isothermal calorimetry is widely used to measure the thermal power and the heat of hydration of cement-based materials. TAM Air (TA Instruments, New Castle, DE, USA) was employed to investigate the effect of NPI on cement hydration. The apparatus was checked and kept at 20 °C. Then, to test the initial baseline, cement pastes were stirred with an electric machine for 2 min, and 14 g of cement paste was transferred into a vial for the test.

#### 2.2.4. Mercury Intrusion Porosimetry (MIP)

The samples were cured for 28 days in water. Then, cement hydration was stopped by ethanol. The samples were vacuum oven-dried for 3 days and were broken into pieces of about 2 g. The MIP technique is extensively applied to characterize the pore structure in porous materials. The testing apparatus used for pore structure measurement is Poremaster GT-60. The MIP test is based on the assumption that the nonwetting liquid mercury (the contact angle between mercury and solid is greater than 90°) will only intrude in the pores of cement-based materials under pressure. The Washburn equation was used to calculate the experimental data.

(1) $$d=\frac{-4\gamma \cos\theta }{P}$$

where

*P* is the pressure of mercury intrusion (N/m^2^),
*d* is the test pore diameter (m),
*γ* is the surface tension of mercury (N/m^2^), and
*θ* is the contact angle between the mercury and the cement paste.

#### 2.2.5. Scanning Electron Microscope

The morphologies of cement hydrates were observed with a Quanta 250 field emission scanning electron microscope (FEI, Hillsboro, OA, USA). An FEI 3D microscope was used. The samples were treated following the same approach applied for MIP and were broken into pieces. Their fresh fracture sections was coated with 15 nm gold before conducting the observation.

#### 2.2.6. Durability Test

The concrete samples were dried for 72 ± 2 h at 105 ± 5 °C, then sealed to cool down for 24 h. The mass of the samples was recorded. Next, the samples were immersed in water, so that 25 ± 5 mm of water was above the tops of the specimens. The water on the sample surfaces was removed with a wet cloth, and the mass of the samples was recorded again. The previous procedure was repeated according to the time schedule. The procedures were consistent with BS 1881-122:2011 Testing concrete. Method for determination of water absorption [36], but the specimens’ dimensions were different: 100 mm × 100 mm × 50 mm. Two samples were used for this test.

Cylindrical concrete was coated with epoxy resin, cut to slices 50 mm thick, and then was vacuum-saturated with a hydroxide solution. Three samples were tested for rapid chloride permeability as per ASTM C1202 [37]. The specimen was fixed in a voltage cell with the positive electrode filled with 0.3 mol/L NaOH solution and—on the opposite side—the negative electrode filled with 3% NaCl solution. Then, a 60 ± 0.1 V direct current was applied across the sample for 6 h. The last total charge passed can be calculated by integrating the current–time data. The three samples were arranged to evaluate the chloride migration coefficient using the rapid chloride migration (RCM) test in line with NT Build 492 [38]. The non-steady-state migration coefficient was mathematically calculated by the following equation:

(2) $$D_{nssm}=\frac{0.0239\left(273+T\right)}{\left(U-2\right)t}\left\{x_{d}-0.0238\sqrt{\frac{\left(273+T\right)Lx_{d}}{U-2}}\right\}$$

where

*D_nssm_* is non-steady-state migration coefficient (×10^−12^ m^2^/s);
*T* is the mean value of the initial and final temperatures in the test anolyte solution (°C);
*L* is the thickness of the test concrete specimen (mm);
*U* is the absolute value of the applied voltage (V);
*t* is the test duration time (h); and
*x_d_* is the average value of the chloride penetration depths (mm).

Two samples were coated with epoxy except the cutting surface, saturated with water, and then immersed in 165 g/L sodium chloride solution. After 57 days, the specimens were carefully ground layer by layer, perpendicular to the exposed surface. The total chloride concentration was obtained through titration, based on the standard NT Build 443 [39].

#### 2.2.7. Contact Angle Test

Three cement pastes were sliced to smooth tablets, then dried at 40 °C for 2 days. The contact angle test of cement paste with water was conducted using Drop Shape Analyzer—DSA25. DSA25 is a reliable instrument for measuring the contact angle and surface energy. The testing range of the contact angle is 1–180°. Cement paste was placed on the stage, and the syringe was adjusted to prepare the maximum water droplet adhering to just the needle tip. The stage was slowly lifted up, and the contact process of the water droplet with the surface of the cement paste was recorded with a high-speed camera. Then, the snapshot was used to analyze the contact angle.

## 3. Results

### 3.1. Isothermal Calorimetry

**Figure 3:**
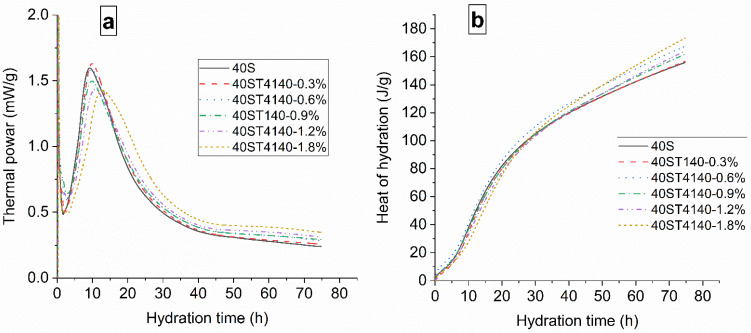
Results of measurements of (**a**) thermal power and (**b**) heat of hydration of cement pastes with 0%, 0.6%, 0.9%, and 1.2% NPI, respectively.

The exothermal rates of cement pastes with different dosages of the NPI were obtained by isothermal calorimetry, as shown in Figure 3. The initial period of cement hydration was prolonged with the addition of the NPI, which may relate to the interaction between the NPI and C_3_A. The induction period of cement hydration was also slightly extended. The exothermal peak of cement hydration was delayed due to the addition of the NPI. However, Figure 3b reveals that the total hydration heat of cement paste was increased by the NPI after 50 h of hydration.

### 3.2. Compressive Strength

**Figure 4:**
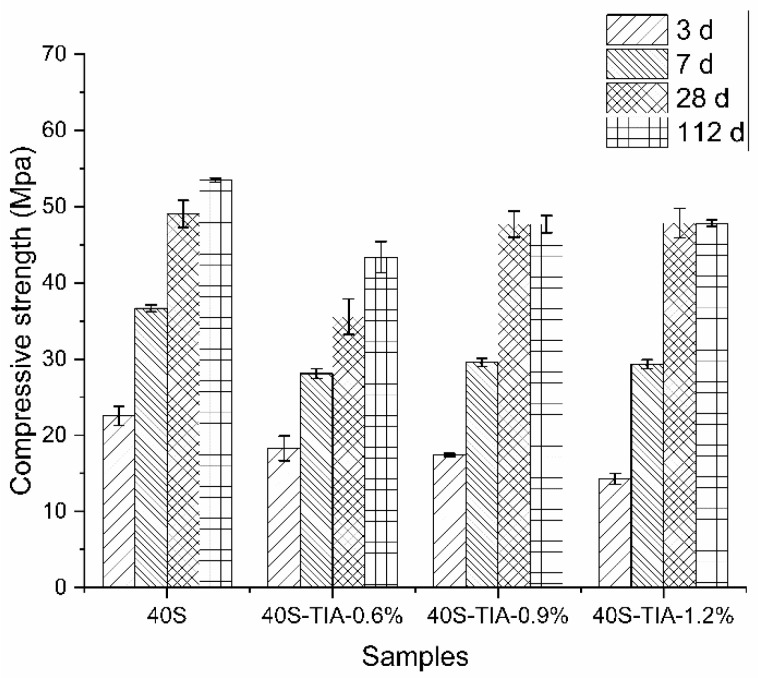
Compressive strength of concrete with different dosages of the NPI at 3, 7, 28, and 112 days, respectively.

Figure 4 shows the compressive strength of concrete incorporated with different dosages of the nano-precursor inhibiting material (NPI) at varied ages. The compressive strength of the concrete samples gradually increased with increasing curing ages for all samples. However, incorporation of the NPI exhibited a negative influence on the development of concrete strength, especially at an early age. At the curing age of 7 days, the compressive strength decreased from about 23 MPa to 12 MPa when the NPI dosage increased from 0.6% to 1.2%. It was also observed that the compressive strength decreased from about 36 MPa to 28 MPa at the age of 14 days. The compressive strength decreased by about 22% to 48% before the age of 28 days. At the curing age of 28 days, the strength decreased from about 49 MPa to 44 MPa when the NPI dosage increased from 0.6% to 1.2%. The compressive strength decrease percentage was restricted to the range of 10% after 112 days.

On the whole, incorporation of the NPI caused a reduction in the compressive strength of concrete. The strength reduction is not significant at the later ages. The compressive strength reduction of the concrete sample incorporated with 0.6% of the NPI was due to a high content of entrained air voids, whereas the hydration was not affected by the NPI, as indicated by the results of isothermal calorimetry.

### 3.3. Water Absorption Ratio

**Figure 5:**
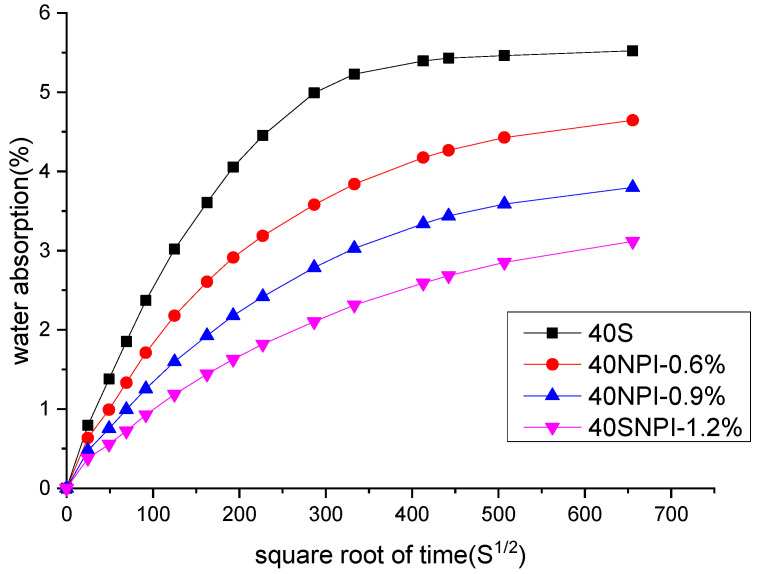
Water absorption of concrete with 0%, 0.6%, 0.9%, and 1.2% NPI respectively.

Figure 5 shows the results of water absorption of concrete incorporated with the NPI from 0% to 1.2%. The results indicated that incorporation of the NPI significantly decreased the water absorption. Besides, the water absorption decreased gradually with increasing incorporation of the NPI. The water absorption of concrete with 1.2% of the NPI decreased by 47% compared with the reference sample. The slope of the water absorption curves at the initial step decreased with increasing incorporation of the NPI, which indicated that incorporation of the NPI contributes to slowing down the speed of water absorption into concrete.

### 3.4. Resistance to Chloride Penetration

**Figure 6:**
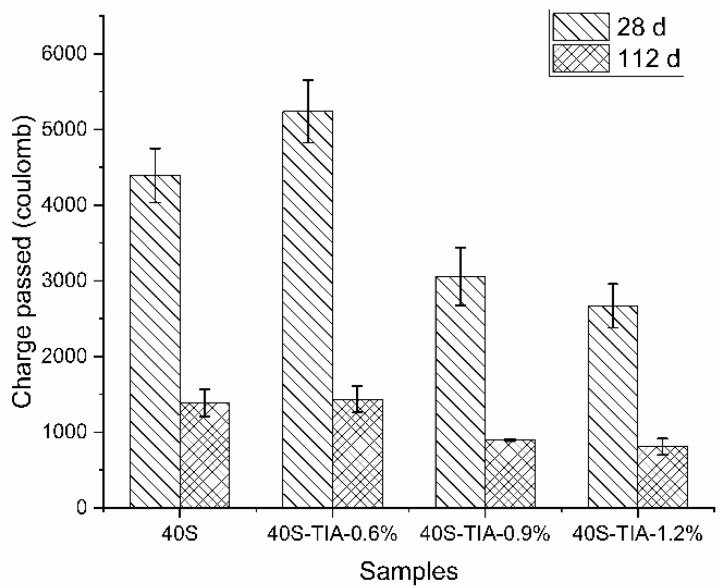
Charge passed through concrete with 0%, 0.6%, 0.9% and 1.2% NPI at 28 days and 112 days, respectively.

Figure 6 shows the rapid chloride penetration test (RCPT) results of concrete. With exception of concrete incorporated with 0.6% NPI, the charge passed decreased with increasing incorporation of the NPI. Besides, the decrease in the charge passed was observed at the age of 112 days, which indicated that the NPI effectively improves the resistance of concrete to chloride penetration. The charge passed through concrete with 1.2% of the NPI decreased from about 4500 coulomb to 2650 coulomb at the age of 28 days, and from about 1400 coulomb to 800 coulomb at the age of 112 days, compared with the reference sample.

**Figure 7:**
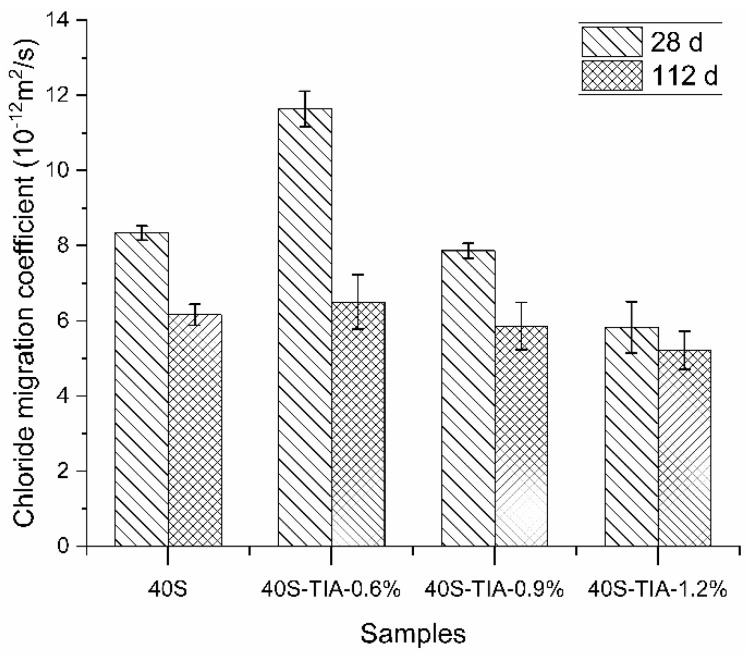
The chloride migration coefficient of concrete with 0%, 0.6%, 0.9%, and 1.2% NPI at 28 days and 112 days, respectively, by RCM.

Figure 7 presents the chloride migration coefficient tested through the rapid chloride migration (RCM) method at 28 days and 112 days, respectively. The migration coefficient of concrete at the age of 28 days decreased when the dosage of the NPI was increased from 0.6% to 1.2%. Compared with the reference sample at the age of 28 days, the chloride migration coefficient of concrete with the NPI decreased from 8.3 × 10^−12^ m^2^/s to 5.9 × 10^−12^ m^2^/s with the exception of concrete with 0.6% dosage of the NPI. The chloride migration coefficient of the concrete sample incorporated with the NPI decreased by 28% to 43% at the curing age of 112 days. It is worth noting that the dosage of 0.6% NPI exhibited the biggest drop compared to other samples. The chloride migration coefficient of the concrete sample decreased from 6.1 × 10^−12^ m^2^/s to 5.2 × 10^−12^ m^2^/s when the dosage of the NPI was increased from 0.6% to 1.2% at the age of 112 days. The concrete samples with 0.6% NPI had higher values of charge passed and the chloride migration coefficient than other groups because of their higher porosity and coarser pore size.

The chloride diffusion coefficient and surface chloride concentration were computed by fitting the relevant data points into Equation (3) by means of a non-linear least-squares regression analysis. The fitting results are listed in Table 3. The incorporation of the NPI decreased the surface chloride concentration from 0.88% to 0.63%. The surface chloride content of the concrete sample with a dosage of 1.2% NPI was the lowest compared with other samples. Besides, the surface chloride concentration of the concrete samples decreased with increasing dosage of the NPI. The NPI incorporation contributed to a reduction in the surface chloride concentration of the concrete sample in a short period of 57 days’ immersion in the salt solution. The significant decrease in the surface chloride concentration could be closely related to the hydrophobic effect of the concrete modified with the NPI.

(3) $$C_{x}=C_{i}+\left(C_{s}-C_{i}\right)\left\{1-erf\left[\frac{x}{2\sqrt{D_{nss}\cdot t}}\right]\right\}$$

where

*C_x_* is the chloride content measured at a certain average depth *x* and exposure time *t* (% by mass of immersed concrete);
*C_s_* is the calculated chloride concentration at the exposed surface (% by mass of concrete);
*C_i_* is the initial chloride content of plain concrete (% by mass of concrete);
*x* is the depth below the top exposed surface to the mid-point of the grounding layer (m);
*D_nss_* is the non-steady state chloride diffusion coefficient (m^2^/s); and
*t* is the exposure time (s).

**Figure 8:**
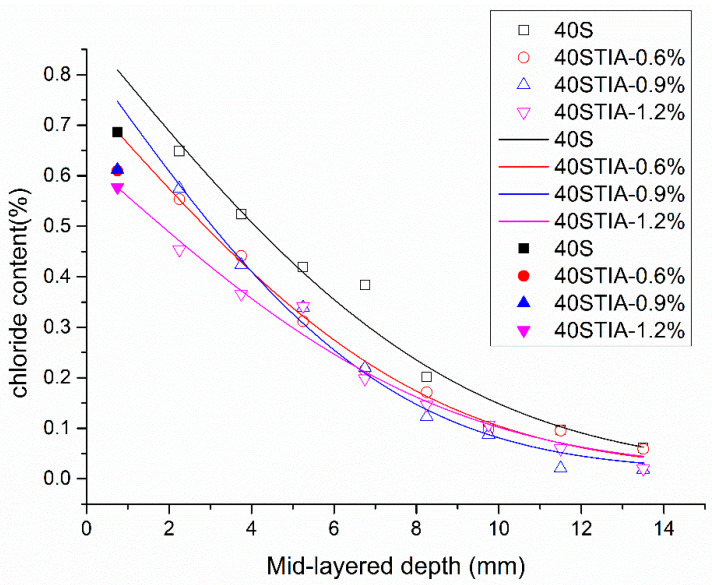
Chloride content and fitting curves of concrete with 0%, 0.6%, 0.9%, and 1.2% NPI, respectively.

Figure 8 displays the chloride concentration and the fitting curves of concrete at different depths immersed in a NaCl solution for 57 days. The chloride concentration of the concrete samples decreased with increasing dosage of the NPI. The NPI effectively decreased the chloride concentration at a depth lower than 5 mm.

The fitting results of the chloride diffusion coefficient were complex. The chloride diffusion coefficient of concrete decreased when the NPI increased from 0% to 0.9%. The chloride diffusion coefficient of concrete with 1.2% NPI increased, but the actual chloride content of the concrete with 1.2% NPI was still the lowest compared with other groups at the same depth. The NPI decreased the chloride diffusivity of concrete. The chloride content was the main factor that resulted in steel corrosion in the concrete structures, so the recommended dosage of the NPI is 1.2% of binder materials.

## 4. Discussion

The NPI contributed to improving the concrete resistance to water and chloride penetration. The role of the NPI on transport properties was ascribed to the optimization of pore structure and increment of hydrophobicity.

### 4.1. Pore Structures

**Figure 9:**
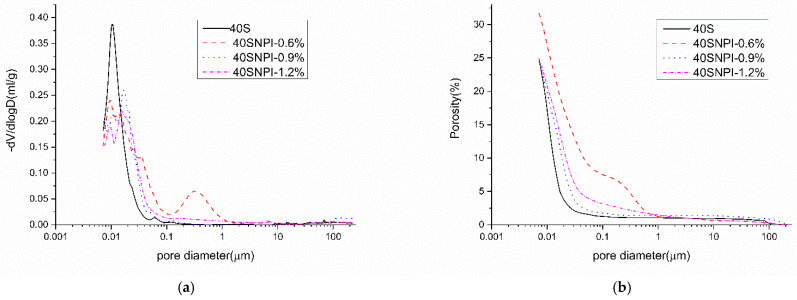
Pore size distribution and the total porosity of cement pastes with 0%, 0.6%, 0.9%, 1.2% NPI tested by MIP. (**a**) Pore size distribution of cement pastes; (**b**) the total porosity of cement pastes.

The results of the pore size distribution and the total porosity of cement paste incorporated with the NPI are shown in Figure 9. The NPI optimized the pore size distribution of hardened cement paste and refined the microstructures of the hardened cement pastes. The peak pore size of cement pastes with the NPI decreased. In addition, the pore sizes below 10 nm changed significantly with an increasing dosage of the NPI.

The results of Figure 9b indicate that incorporation of 0.9% and 1.2% NPI effectively decreased the porosity of hardened cement paste for a pore size lower than 0.02 μm, but slightly increased the porosity for the pore size higher than 0.02 μm. As for the sample 40SNPI-0.6%, it seems that high entrained air content due to low dosage of NPI resulted in the increased porosity for a pore size higher than 0.2 μm, which also explained the decrease in compressive strength in Figure 4. The large capillary pores (range between 10 μm ≥ d ≥ threshold pore diameter) were mostly disconnected and had a minor effect on the ion transport property according to Zhang and Ye’s results [40]. As for our research, the effect of minor addition of pore sizes over 10 μm could not be significant. A pore size change below 100 nm would play a more prominent role in the transport of chloride. The RCPT and RCM results showed that resistance of chloride penetration was promoted.

According to Table 4, the NPI did not change the cumulative pore volume of cement pastes except the cement specimen with 0.6% NPI. However, some literature reports that hydrophobic chemicals increase the total porosity of cement pastes. For instance, Feng et al. [41] reported that the incorporation of hydrophobic vinyl-based silane slightly increased the total porosity compared with that of the control sample, which was related to the homogeneous distribution of cement hydrated products that caused smaller capillary porosity. Zhu et al. [42] found that a water-repellent additive can improve the pore structure of concrete by partly or completely filling the existing pore channels. This additive was produced by treating mica powder surface with a silane coupling agent and polydimethylsiloxane. In addition, mixing stearic acid aqueous emulsion into cement paste also increased the porosity [21]. The NPI had both hydrophilic groups and a long hydrophobic carbon chain compared with the reported vinyl triethoxysilane and stearic acid aqueous emulsion. The hydrophilic groups of the NPI interacted with cement hydrates, and the long hydrophobic carbon chain of the NPI spread on the surface and repelled the agglomerate of hydrated particles, while the total porosity of cement pastes incorporated with 0.9% and 1.2% NPI changed slightly compared with the reference sample. The reason was the hydrophilic groups reacting with portlandite to optimize the pore structures of the cement pastes, which compensated for the retardation effect of the long hydrophobic carbon chains on cement hydration.

### 4.2. Contact Angle

**Figure 10:**
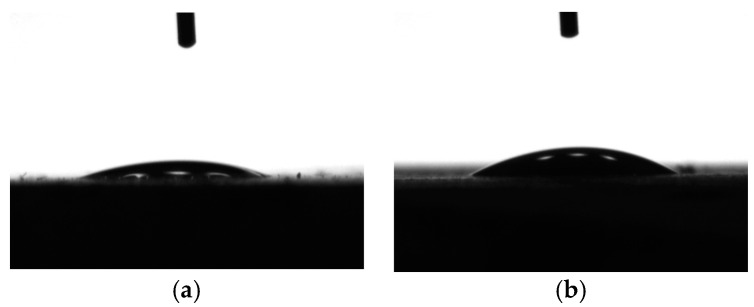

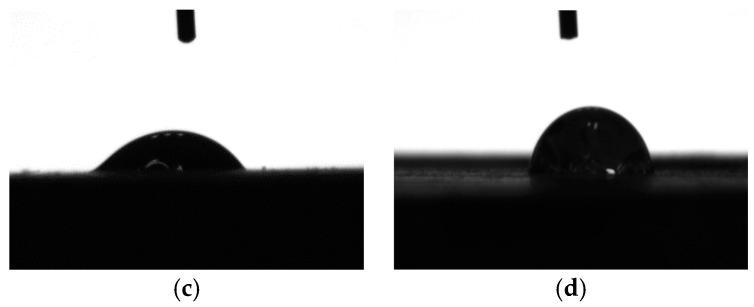
Images of the contact angle of cement pastes with 0% (**a**), 0.6% (**b**), 0.9% (**c**), and 1.2% NPI (**d**).

As mentioned at the beginning of the present research, the NPI can increase the hydrophobicity of concrete to resist water absorption. The results of the contact angles of cement pastes modified with the NPI are listed in Table 5. The contact angle gradually went up with increasing dosage of the NPI tested at 5 s and 30 s. Figure 10 presents the images of the contact angles of cement pastes with 0%, 0.6%, 0.9%, and 1.2% NPI, respectively. Their corresponding contact angles were 17.8°, 19.2°, 39.3°, and 85.8° after the water droplet contacted the surface of the cement paste for 5 s. Atzeni et al. [13] also observed similar results. The contact angles of cement pastes treated with silicic esters or alkylalkoxysiloxane were all below 90°. The contact angle of cement pastes treated with SiO_2_/polymethylhydrosiloxane was 116.5° according to Li et al. [9]. The molecular dynamics results from Hou et al. [12] showed that the silane molecule structure bridged on the calcium silicate hydrate, which made hydrophilic CSH surface hydrophobic, so that the hydrophobic silane coating films could inhibit water penetration into the nanometer pores of CSH gel. The NPI interacted with cement hydrates and changed the hydrophilicity of the cement pastes, which was confirmed by contact angles. Considering the results of isothermal calorimetry, the compressive strength, and the contact angle, the NPI mainly impacted the interface properties of hydrated products.

The NPI not only optimized the pore structures of cement pastes but also increased hydrophobicity. Therefore, incorporation of the NPI was helpful to increase the capacity of concrete to resist the transport of aggressive ions and water, which was proved through the results of water absorption, chloride migration, and diffusivity described in the present research.

### 4.3. Morphology of Cement Paste

**Figure 11:**
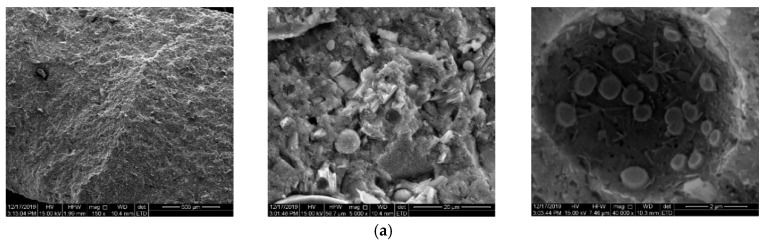

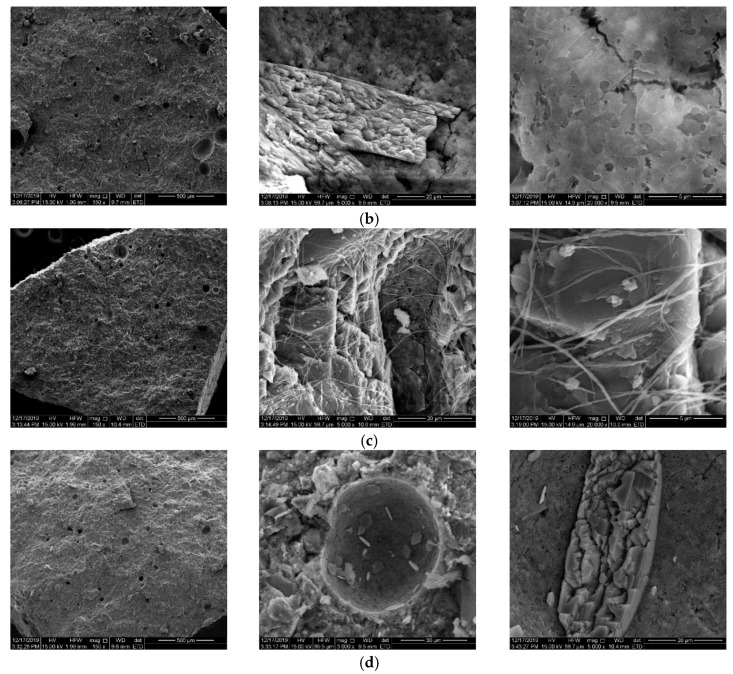
SEM pictures of cement pastes with different dosage of the NPI: (**a**) 0%, (**b**) 0.6%, (**c**) 0.9%, and (**d**) 1.2%.

It was reported [43,44] that the usage of organic polymers had a significant influence on the morphology of portlandite in cement paste. Figure 11 shows the morphologies of cement paste with different dosages of the NPI. The NPI slightly decreased portlandite, and the morphology of portlandite became untypical. There were smooth and porous materials around the big pores, and the NPI entrained a few micrometer-size pores, especially for samples with 0.6% NPI, for which the total porosity was also the highest. The transport property of materials was not only controlled by the porosity but also by the connectivity of pores. Comparing the results of the MIP test and chloride content in concrete, it can be inferred that the NPI inhibits the transport of chloride by decreasing the connectivity of pores and/or forming hydrophobic films in cement pastes.

## 5. Conclusions

A novel nano-precursor inhibiting material (NPI), organic carboxylic acid ammonium salt, was used to modify the properties of concrete. It was a promising material for increasing the hydrophobicity of cement paste to improve the resistance of water and chloride ion penetration into concrete, meanwhile minimizing the negative effect of the hydrophobic hydrocarbon chain on the compressive strength of concrete.

(1) The NPI caused a reduction in the strength of concrete, but the strength reduction was minor at a later age. The NPI increased the total porosity and entrained big capillary pores in cement pastes, and the alkane chain resulted in a weaker ITZ in concrete. These reasons led to a decline in the compressive strength of the concrete samples.
(2) The incorporation of the NPI significantly decreased water absorption and slowed down the speed of water sorptivity in concrete. The NPI also decreased the charge passed and the chloride migration coefficient of concrete. It is worth noting that the NPI effectively decreased the chloride diffusion coefficient and the chloride content at a depth lower than 5 mm and the surface chloride concentration of concrete.
(3) The improvement in the transport properties of concrete was due to the incorporation with the NPI, which resulted in a gradual ascent of the contact angle from 17.8° to 85.8° when the dosage of the NPI was increased from 0% to 1.2%, and the surfaces of cement paste became less hydrophilic. Moreover, the NPI also changed the pore size distribution of hardened cement paste.

## Figures and Tables

**Table 1:** Chemical composition of reference cement, fly ash, and slag (mass%).

Oxide	Cement	Fly Ash	Slag
CaO	61.75	7.40	41.5
SiO_2_	20.64	43.9	32.2
Al_2_O_3_	4.62	34.8	14.6
Fe_2_O_3_	2.82	6.13	0.96
K_2_O	0.48	1.09	0.57
MgO	2.06	0.65	6.37
Na_2_O	0.12	0.43	0.30
SO_3_	1.20	2.00	2.12
TiO_2_	0.29	1.51	0.61

**Table 2:** Mix design of concrete (kg/m^3^).

Sample	Cement	Slag	Fly Ash	Water	Sand	Fine Aggregate	Coarse Aggregate	NPI
40S	269.5	147	73.5	196	703	405	607	0
40SNPI-0.6%	269.5	147	73.5	180	703	405	607	16
40SNPI-0.9%	269.5	147	73.5	172	703	405	607	25
40SNPI-1.2%	269.5	147	73.5	163	703	405	607	33

**Table 3:** The regression analysis results of chloride concentration in the concrete samples.

Mark	*C_S_*	*D_nss_* (10^−12^ m^2^/s)	R^2^
40S	0.88	4.96	0.97
40SNPI-0.6%	0.76	4.18	0.99
40SNPI-0.9%	0.83	3.29	0.99
40SNPI-1.2%	0.63	4.63	0.97

**Table 4:** The total porosity and the mean pore size of cement pastes calculated by MIP.

Mark	Porosity (%)	Mean Pore Size (nm)
40S	24.7	16.1
40SNPI-0.6%	31.7	20.0
40SNPI-0.9%	24.5	15.2
40SNPI-1.2%	24.9	17.2

**Table 5:** Contact angle of cement pastes.

Sample No.	Contact Angle (°), T = 5 s	Contact Angle (°), T = 30 s
40S	17.8 ± 1.2	9.8 ± 0.15
40SNPI-0.6%	19.2 ± 0.7	17.3 ± 0.4
40SNPI-0.9%	39.3 ± 2.2	34.3 ± 4.6
40SNPI-1.2%	85.8 ± 2.2	78.2 ± 0.9
